# Behavioral outcome of very preterm children at 5 years of age: Prognostic utility of brain tissue volumes at term‐equivalent‐age, perinatal, and environmental factors

**DOI:** 10.1002/brb3.2818

**Published:** 2023-01-14

**Authors:** Maria Chiara Liverani, Serafeim Loukas, Laura Gui, Marie‐Pascale Pittet, Maricé Pereira, Anita C. Truttmann, Pauline Brunner, Myriam Bickle‐Graz, Petra S. Hüppi, Djalel‐Eddine Meskaldji, Cristina Borradori‐Tolsa

**Affiliations:** ^1^ Division of Development and Growth, Department of Pediatrics University of Geneva Geneva Switzerland; ^2^ Sensorimotor, Affective and Social Development Laboratory, Faculty of Psychology and Educational Sciences University of Geneva Geneva Switzerland; ^3^ Institute of Bioengineering, Ecole Polytechnique Fédérale de Lausanne (EPFL) Lausanne Switzerland; ^4^ Clinic of Neonatology, Department of Women Mother Child University Center Hospital and University of Lausanne Lausanne Switzerland; ^5^ Follow Up Unit, Department of Women Mother Child University Center Hospital and University of Lausanne Lausanne Switzerland; ^6^ Institute of Mathematics, Ecole Polytechnique Fédérale de Lausanne (EPFL) Lausanne Switzerland

**Keywords:** behavioral outcome, classification, machine learning, MRI, preterm infants, volumetric brain data

## Abstract

**Objective:**

Prematurity is associated with a high risk of long‐term behavioral problems. This study aimed to assess the prognostic utility of volumetric brain data at term‐equivalent‐age (TEA), clinical perinatal factors, and parental social economic risk in the prediction of the behavioral outcome at 5 years in a cohort of very preterm infants (VPT, <32 gestational weeks).

**Methods:**

T2‐weighted magnetic resonance brain images of 80 VPT children were acquired at TEA and automatically segmented into cortical gray matter, deep subcortical gray matter, white matter (WM), cerebellum (CB), and cerebrospinal fluid. The gray matter structure of the amygdala was manually segmented. Children were examined at 5 years of age with a behavioral assessment, using the strengths and difficulties questionnaire (SDQ). The utility of brain volumes at TEA, perinatal factors, and social economic risk for the prediction of behavioral outcome was investigated using support vector machine classifiers and permutation feature importance.

**Results:**

The predictive modeling of the volumetric data showed that WM, amygdala, and CB volumes were the best predictors of the SDQ emotional symptoms score. Among the perinatal factors, sex, sepsis, and bronchopulmonary dysplasia were the best predictors of the hyperactivity/inattention score. When combining the social economic risk with volumetric and perinatal factors, we were able to accurately predict the emotional symptoms score. Finally, social economic risk was positively correlated with the scores of conduct problems and peer problems.

**Conclusions:**

This study provides information on the relation between brain structure at TEA and clinical perinatal factors with behavioral outcome at age 5 years in VPT children. Nevertheless, the overall predictive power of our models is relatively modest, and further research is needed to identify factors associated with subsequent behavioral problems in this population.

## 1 INTRODUCTION

Preterm birth is associated with high rates of long‐term morbidities and a broad spectrum of neurodevelopmental impairments (Pierrat et al., 2021). Several studies have shown that preterm birth can impact the ability to regulate emotions, tolerate frustration, and modulate behavior in different situations (Bora et al., 2011; Johnson & Marlow, 2011), resulting in impaired social skills as well as cognitive and behavioral difficulties. The “preterm behavioral phenotype,” well described by Johnson and Marlow (2011), is characterized by a triad of inattention symptoms, social difficulties, and emotional problems that may affect educational attainment and quality of life.

Behavioral problems in children and adolescents are often assessed through questionnaires. The strength and difficulties questionnaire (SDQ) is a frequently used and well‐validated screening tool investigating behavioral problems in subjects aged from 4 to 16 (Goodman, 1997). It is short to fill in and has good psychometric properties in detecting mental health problems. The SDQ was used to investigate the behavioral profile of children born preterm (Johnson et al., 2014; Pierrat et al., 2021), confirming the increased risk for social difficulties, emotional and attention problems, and symptoms related to the triad described by Johnson and Marlow (2011).

Several research studies have shown that specific factors such as sex, neonatal characteristics and morbidities, and family environment are risk factors for later development, and that the cumulative effect of biological risk and family social risk can impact the cognitive and behavioral development of very preterm (VPT) children (Ritchie et al., 2015; Treyvaud et al., 2013).

Even without clear brain injury, preterm birth is often characterized by subtle alterations of cerebral structures, leading to potential disruption in the typical progression of children's development and to subsequent functional consequences. Compared to term‐born babies, preterm newborns show abnormal tissue volumes at term‐equivalent‐age (TEA; Inder et al., 2005; Keunen et al., 2012; Peterson et al., 2003). These alterations can persist until childhood and adolescence (de Kieviet et al., 2012).

Some studies have investigated the relationship between structural brain alterations at TEA and neurodevelopmental outcome in children born too soon (Gui et al., 2019; Keunen et al., 2016; Moeskops et al., 2017). A recent study demonstrated a relationship between brain tissue volumes at TEA and early motor behavior in VPT infants (Katušić et al., 2020). In addition, Lind et al. (2010) demonstrated significant associations between smaller volumes of total brain tissue and poorer executive functions, between smaller cerebellar volumes and both poorer executive functions and motor skills, as well as between larger brainstem volume and poorer language functions. Similarly, Gui et al. (2019) showed that brain tissue volumes at TEA and volume growth rates between birth and TEA contributed somewhat to the prediction of the cognitive outcome at 5 years of age. Finally, neonatal brain abnormalities have been associated with psychiatric diagnoses and socio‐emotional problems in school‐aged VPT children (Treyvaud et al., 2013). Such studies might offer indications as to what extent neonatal brain magnetic resonance imaging (MRI) can represent a valuable instrument for predicting developmental outcomes in this vulnerable population. Nevertheless, it should be noted that there are also reviews in which no statistically significant associations between brain volumes measured at TEA and the outcome of preterm children have been found (Anderson et al., 2015; Keunen et al., 2012).

Only a few studies have investigated possible relationships between brain volumes at TEA and subsequent behavioral outcome (Rogers et al., 2012). Therefore, the main objective of this research was to evaluate the ability of brain volumes at TEA to predict behavioral outcome assessed by the SDQ at 5 years of age in VPT children (born <32 gestational weeks). Additionally, we investigated the contribution of perinatal factors and parental social economic risk to the prediction of behavioral outcome at this age.

## 2 METHODS

### 2.1 Cohort

Among children born below 32 gestational weeks at 2 level III NICUs (University Hospitals of Geneva and Lausanne) between 2007 and 2013, we included in our analysis only those who underwent a MRI exam at TEA and had a cognitive and behavioral assessment at 5 years of age (*n* = 86). MRI quality was not acceptable for six participants who were excluded from the analysis. The final sample included 80 VPT children. Exclusion criteria included severe physical or sensory disabilities, chromosomal abnormalities, and the presence of severe intraventricular hemorrhage with ventricular dilatation or hemorrhagic infarction identified by sequential US and MRI at TEA. Gestational age (GA) was based on the last menstrual period or the best estimation from a prenatal ultrasound. Birthweight (BW) *z*‐scores were calculated based on the growth curves by Voigt et al. (2006). The presence of proven sepsis was defined as at least one positive blood culture during the hospital stay. Bronchopulmonary dysplasia (BPD) was defined as the need for supplemental oxygen or ventilatory support at 36 weeks postmenstrual age (Jobe & Bancalari, 2001). Parental social economic risk was estimated using the Largo scale, which results in a score (=SES) ranging from 2 to 12, based on maternal education and paternal occupation. The higher the social economic score (SES) the higher the parental social economic risk (Largo et al., 1989). Data collection was approved by the Ethics Committees. The research was carried out according to the Declaration of Helsinki.

### 2.2 MRI acquisition

The MRI scan was performed at TEA (GA at scan: 40.1 ± 0.8 weeks, range: 38.3–41.9 weeks) during infants’ natural sleep. Infants were positioned inside the scanner, wrapped in a vacuum pillow, and monitored with electrocardiography and pulse oximetry. Earmuffs were used for noise attenuation. Imaging data were acquired at both hospitals using identical Siemens Trio Tim (3T) MRI scanners and protocols. T2‐weighted images were acquired using the same protocol at both sites a turbo spin‐echo sequence with parameters: TE = 150 ms, TR = 4600 ms, voxel size: 0.8 × 0.8 × 1.2 mm^3^.

### 2.3 Image processing

The automatic segmentation method of Gui et al. (2012) was employed to segment all scans into cortical gray matter (CGM), white matter (WM), deep subcortical gray matter (DSGM), cerebellum (CB), and cerebrospinal fluid (CSF). The DSGM included: thalamus, hypothalamus, globus pallidus, dorsal striatum (putamen, caudate), ventral striatum (nucleus accumbens), and subthalamic nucleus. The resulting segmentations were visually examined, and minor manual corrections were performed when necessary. Tissue volumes were then automatically extracted from the final segmentations. Amygdalae were manually segmented in each scan, and their volumes were calculated using ITK‐SNAP software. Segmentation was performed on T2‐weighted images by a physician trained in this task for 6 months.

### 2.4 Behavioral and cognitive outcome

All children were followed up around their fifth birthday and had a neurological examination and an assessment of cognitive outcome performed by developmental pediatricians and psychologists. Behavior was assessed with the SDQ, parental report (Goodman, 1997), a 25‐item questionnaire composed of 5 subscales assessing *emotional symptoms*, *conduct problems*, *hyperactivity/inattention*, *peer problems*, and *prosocial behavior*. For each item, parents are asked to answer on a 3‐point scale (0 = *not true*, 1 = *somewhat true*, 2 = *certainly true*). The score for each subscale is the sum of the related items with higher scores reflecting more serious difficulties in the domain, except for the *prosocial behavior* subscale, for which a high score means positive behavior. A *total difficulties score* is calculated by adding all the subscales scores except the *prosocial behavior* one. The official scoring guidelines (http://www.sdqinfo.org) classify the total difficulties score and each subscale as “normal,” “borderline,” or “abnormal.” Given that most of our participants scored in the normal range (*normal group*), we pooled together children with borderline and abnormal scores (*at‐risk group*). Intelligence was assessed with the French version of the Kaufman Assessment Battery for Children (Kaufman & Kaufman, 2004). This psychological diagnostic test allows the calculation of cognitive scores, including a summary score, the composite mental processes, which is considered equivalent to an intelligence quotient and has an expected mean of 100 and a standard deviation (SD) of 15.

### 2.5 Statistical analysis

*Predictive modeling*: The present study includes three sets of variables: the volumetric data (DSGM, CGM, WM, CB, CSF, amygdala), the perinatal factors (GA, BW *z*‐score, sex, BPD, sepsis), and the parents’ social economic risk. To investigate the predictive ability of these sets of variables for the identification of children with normal or at‐risk behavioral outcomes (SDQ subscales) at 5 years of age, we employed support‐vector‐classifiers (linear‐SVC, default parameters). The prosocial subscale of the SDQ was not used as all participants were categorized into the normal group. Classifications were performed using different combinations of the three sets of features mentioned above: (i) the volumetric data, (ii) the perinatal factors, and (iii) all three sets together. The SES as an environmental factor was considered separately to unveil its contribution to the perinatal factors in terms of predictive power. Stratified fivefold cross‐validation, with internal standardization of the features (*μ* = 0, *σ* = 1), was considered to avoid over‐fitting, bias introduction from different variable scales issues, and to deal with the unbalanced dataset (Poldrack et al., 2020). The predictability was assessed using receiver operating characteristic (ROC) curves. Finally, to quantify the feature importance of the input variables, we employed a permutation‐based method known as mean decrease accuracy (MDA) analysis using 1000 permutations (Breiman, 2001), which assesses the importance of a variable by measuring the decrease in the classification accuracy when the variable is shuffled.

*Linear models*: As a final post hoc step, we explored the association between the SES score and the SDQ subscales.

## 3 RESULTS

### 3.1 Cohort characteristics and brain tissue volumes at TEA

Table 1 illustrates the perinatal characteristics of our cohort, derived from medical records, together with the average values of the absolute brain volumes measured at TEA.

**TABLE 1:** Cohort characteristics

Perinatal data	
Male gender, n (%)	36 (45)
GA, weeks, mean (SD), range	27.79 (1.64) 23.86 – 31
BW, g, mean (SD), range BW z‐score, mean (SD)	989 (231.93) 600 ‐ 1530 −0.4 (0.71)
BPD, n (%)	30 (37.5)
Sepsis, n (%)	23 (28.75)
PVL, n (%)	2 (2.5)

Parental socio‐economic risk	
SES score, mean (SD), range	6.43 (2.96) 2 ‐ 12

Absolute tissue volumes at TEA, ml	
CGM, mean (SD)	165.78 (23.59)
DSGM, mean (SD)	21.13 (1.97)
WM, mean (SD)	147.80 (15.94)
Amygdala, mean (SD)	0.82 (0.15)
CB, mean (SD)	22.71 (3.61)
CSF, mean (SD)	100.89 (25.51)

Behavioural outcome at 5 years (SDQ)	
Total difficulties Score *Normal (range 0‐13)* *At risk (range 14‐40)* Emotional symptoms score *Normal (range 0‐3)* *At risk (range 4‐10)* Conduct problems score *Normal (range 0‐2)* *At risk (range 3‐10)* Hyperactivity/inattention score *Normal (range 0‐5)* *At risk (range 6‐10)* Peer problems score *Normal (range 0‐2)* *At risk (range 3‐10)* Prosocial behaviour score *Normal (range 6‐10)* *At risk (range 5‐0)*	69 (86%) 11 (14%) 63 (79%) 17 (21%) 61(76%) 19 (24%) 66 (83%) 14 (17%) 66 (83%) 14 (17%) 80 (100%) 0 (0%)

*Note*: Perinatal data, parental socioeconomic risk, brain volumes, and behavioral outcome at 5 years of our cohort.

Abbreviations: BPD, bronchopulmonary dysplasia; BW, birthweight; CB, cerebellum; CGM, cortical gray matter; CSF, corticospinal fluid; DSGM, deep subcortical gray matter; GA, gestational age; PVL, periventricular leukomalacia; SD, standard deviation; SDQ, strengths and difficulties questionnaire; SES, social economic score; TEA, term‐equivalent‐age; WM, white matter.

Two children had a small unilateral cystic periventricular leukomalacia confirmed by MRI at TEA but no other WM lesion, so they were not excluded from the study.

### 3.2 Outcome at 5 years of age

**FIGURE 1:**
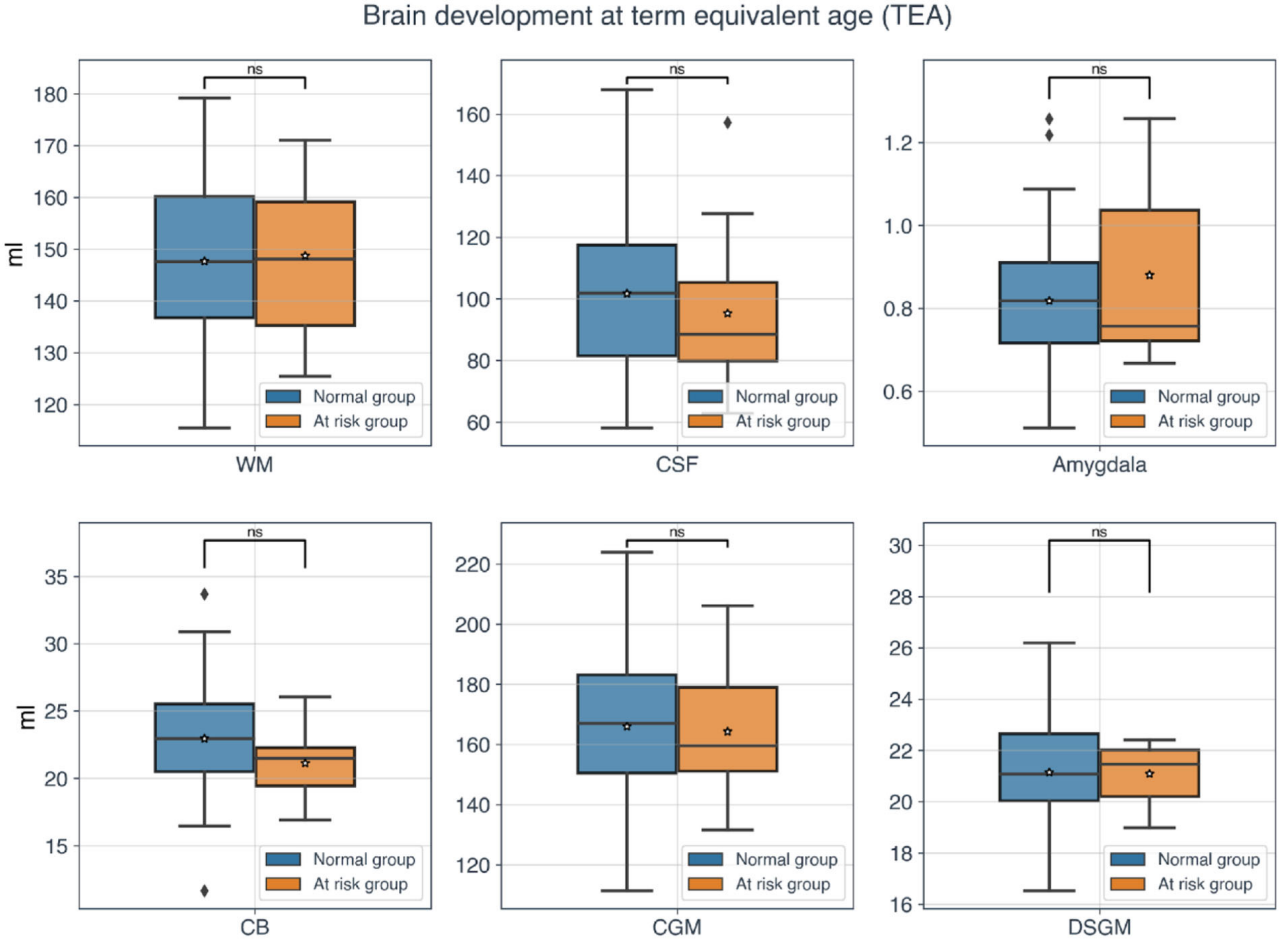
Brain absolute volumes at term‐equivalent‐age (TEA). Boxplots for white matter (WM), cerebrospinal fluid (CSF), amygdala, cerebellum (CB), cortical gray matter (CGM), and deep subcortical gray matter (DSGM) of the two groups of participants based on the strengths and difficulties questionnaire (SDQ) total difficulties score variable. Blue and orange colors represent the normal and at‐risk group, respectively. No statistically significant differences between the volumetric data of the two groups were observed. The asterisk represents the mean value, whereas the black line inside the boxplots depicts the median.

Mean age at follow‐up was 62 months (SD 2.6). Eighty percent of children had a composite mental processes score in the normal range (i.e., score >85, as assessed by the K‐ABC battery). Table 1 shows the scores of the SDQ questionnaire for the total difficulties score and each subscale. The majority of the participants had a normal total difficulties score. The behavioral problems that were mostly represented in our population were conduct problems and emotional symptoms (23% and 20%, respectively), followed by a relatively low rate of peer problems and hyperactivity/inattention symptoms (17% and 17%, respectively). Concerning the prosocial behavior subscale, all participants scored within the normal range. No significant differences were observed for any brain absolute tissue volumes (CGM, DSGM, CB, WM, CSF, amygdala) between the normal and at‐risk SDQ groups (see Figure 1).

### 3.3 Predictive modeling and classification of the behavioral outcomes

**FIGURE 2:**
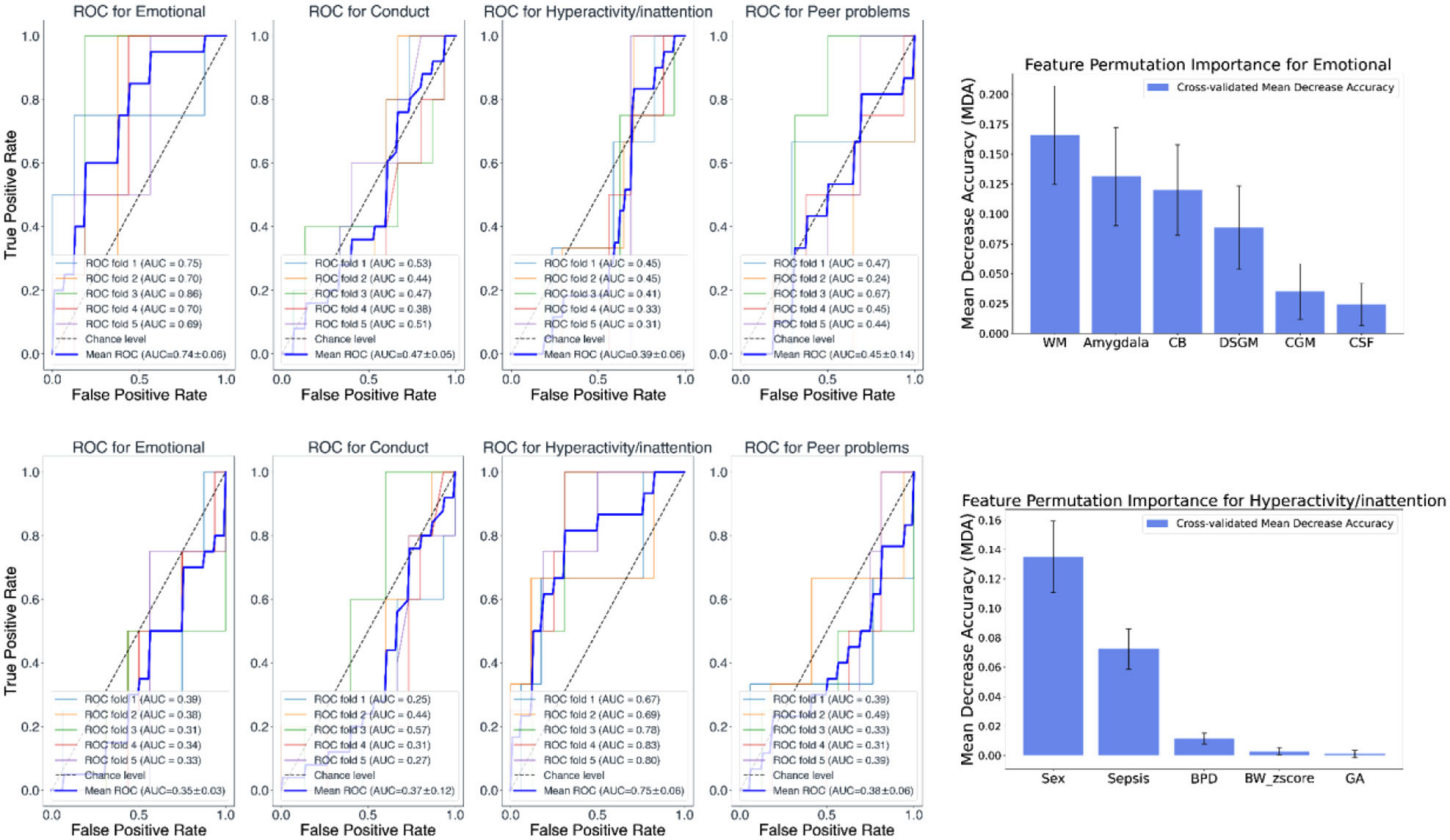
Top panel (left): Classification receiver operating characteristic (ROC) curves using only the volumetric data and to predict the four strengths and difficulties questionnaire (SDQ) subscales (i.e., emotional, conduct, hyperactivity/inattention, and peer problems). Top panel (right): Mean decrease accuracy (MDA) values of the volumetric variables. Bar height represents the average across permutations MDA value, and the error bars show the standard deviation of these MDA values. Bottom panel shows similarly, the results when using only the perinatal factors as predictors in the classification model.

*Volumetric data*: The top left panel of Figure 2 shows the ROC curves with cross‐validation when only the volumetric data were used as features. Only the performance of the model predicting the emotional symptoms subscale is reliable, with an average area under the curve (AUC) 0.74 (0.06) across all the stratified folds. The top right panel of Figure 2 indicates that for this model, the most informative features are the WM, amygdala, CB, DSGM, CGM, and CSF volumes. The remaining models have an average AUC around chance level, indicating that those models are not reliable.

*Perinatal clinical factors*: We then aimed to investigate to which extent perinatal factors were predictors of the behavioral outcome. The bottom left panel of Figure 2 shows the classification ROC curves when only the perinatal factors were used as features. Only the performance of the model predicting the hyperactivity/inattention subscale is reliable, with an average AUC 0.75 (0.06). The bottom right panel of Figure 2 indicates that the most informative features in terms of classification performance for this model are sex and sepsis. On the other hand, the BW *z*‐score and GA variables are not considered important in terms of predictions as the range of the SD of the MDA values includes negative values. Furthermore, it is worth pointing out that the MDA values are small, meaning that the influence on the model's predictive power is limited. Post hoc analysis showed that females tend to have slightly higher levels of hyperactivity/inattention, although the difference is not statistically significant (*p* = .82). Similarly, children with sepsis showed higher levels of hyperactivity/inattention compared to children without sepsis, even if the comparison felt short of significance (*p* = .18).

**FIGURE 3:**
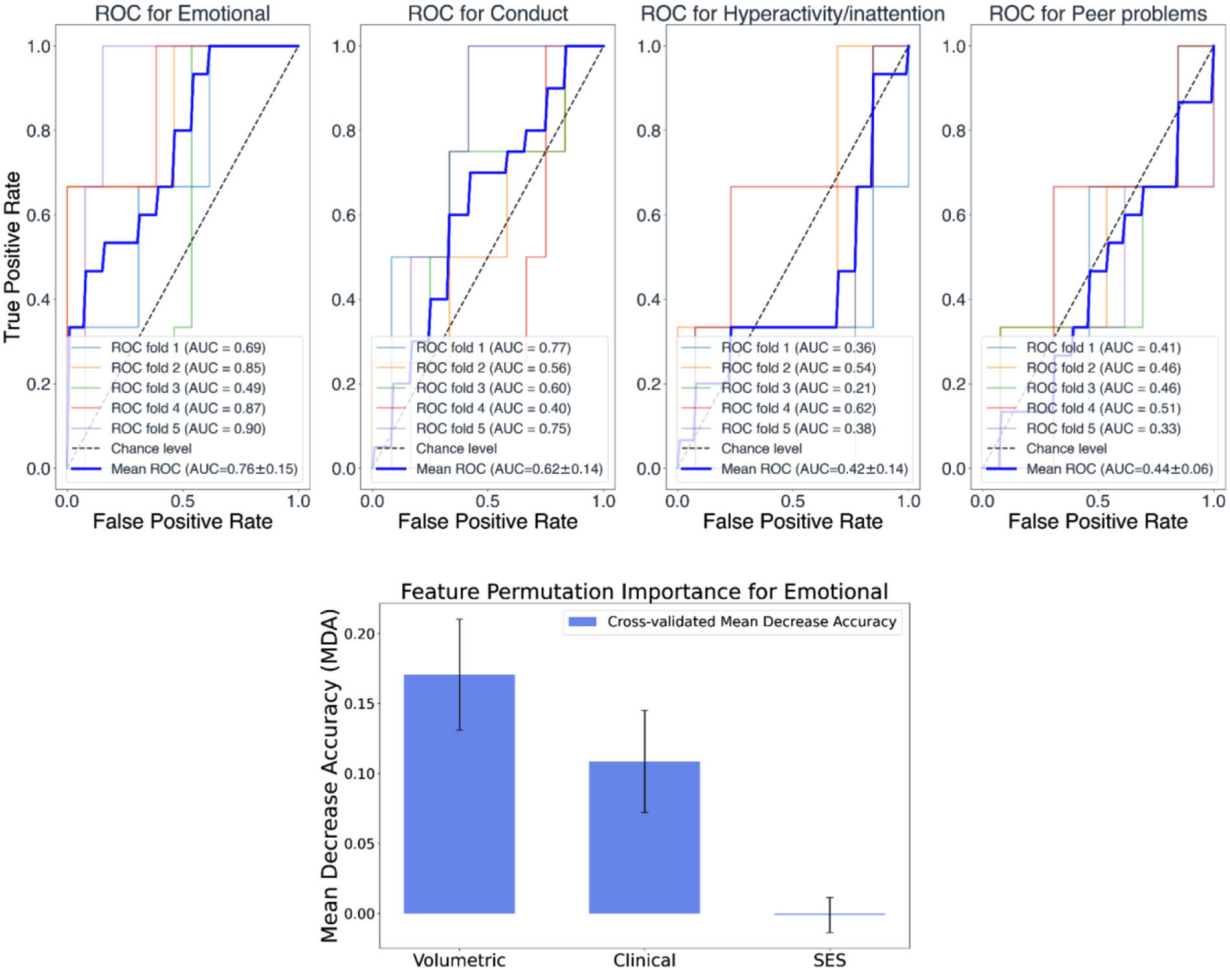
Top panel: Classification receiver operating characteristic (ROC) curves using all three sets of variables (volumetric, perinatal, and social economic score [SES]) and, as target variables, the four strengths and difficulties questionnaire (SDQ) subscales (i.e., emotional, conduct, hyperactivity/inattention, and peer problems). Bottom panel: Mean decrease accuracy (MDA) values of the individual sets of variables. Bar height represents the average across permutations MDA value for the specific set of variables, and the error bars show the standard deviation of these MDA values.

*All three sets of variables*: The top panel of Figure 3 shows the classification ROC curves with cross‐validation when all three sets of variables were used as features (volumetric, perinatal, and SES). The performance of the models predicting the emotional symptoms subscale is reliable, with an average AUC of 0.76 (0.15). The bottom panel of Figure 3 indicates that the most informative set of features in terms of classification performance for the emotional symptoms subscale model is the volumetric set of variables. The remaining models were not reliable and thus, not further explored.

### 3.4 Post hoc analysis for the SES variable

Post hoc analysis with linear models showed that a high parental social economic risk was associated with a higher conduct problems score (*r* = .155, *p* = .0015), as well as a higher peer problems score (*r* = .156, *p* = .0098), but not with any other behavioral scores.

## 4 DISCUSSION

In this study, we investigated the ability of brain tissue volumes measured at TEA, clinical perinatal factors, and parental social economic risk to predict behavioral outcome at 5 years of age in VPT children.

The SDQ is one of the most popular questionnaires used to assess behavioral problems in children and adolescents, showing very high reliability and acceptance among parents (Bartal et al., 2020; Johnson et al., 2014). In our sample, 86% of VPT children showed a total difficulties score in the normal range. Abnormal scores were mainly found in the conduct problems subscale (24%), followed by emotional symptoms (21%). Finally, relatively low rates of peer problems and hyperactivity/inattention symptoms have been found in our sample (17%). These results are partly consistent with those of a recent paper on a Swiss cohort of VPT children of the same age (Bartal et al., 2020), where emotional problems were reported to occur more frequently compared to term‐born children, and hyperactivity/inattention was not a prominent symptom. Other studies using the SDQ found a higher occurrence of conduct problems and emotional symptoms in this clinical population (Fevang et al., 2017; Johnson et al., 2014). These studies also reported a higher rate of hyperactivity/inattention symptoms and peer problems, which is not in‐line with our findings. The low prevalence of hyperactivity/inattention symptoms detected by the SDQ in our participants may be related to the fact that preterm birth appears to be associated with a higher risk of the inattentive subtype of the attention deficit hyperactivity disorder, than hyperactivity. In young children, parents report symptoms of hyperactivity more often than inattention, raising the question of whether the SDQ detects symptoms of hyperactivity better than inattention.

A growing body of research has focused on the utility of volumetric features in predicting neurodevelopmental outcomes. Nevertheless, there is still no consensus, especially with regard to the prediction of behavioral and emotional aspects of children's development. Therefore, to study the relationship between brain tissue volumes measured at TEA and subsequent behavioral outcome, we performed a predictive analysis using SVC models to classify subjects into normal and at‐risk.

Using the volumetric dataset, we could predict the emotional symptoms’ score with a satisfying AUC = 0.74. Permutation importance analysis revealed that the most important variables, in terms of classification accuracy, were the WM, the amygdala, and the CB absolute volumes. These findings are consistent with previous studies, in which poorer socio‐emotional outcomes in children born preterm have been associated with structural brain alterations in regions related to emotional processing, such as reduced volumes of the amygdala (Cismaru et al., 2016), CB (Montagna & Nosarti, 2016), and hippocampus (Peterson, 2000). Recent studies also confirmed the association between WM aberration and emotional disorders, showing microstructural abnormalities in cingulum‐callosal WM pathways and uncinate fasciculus in children with emotional dysregulation (Hung et al., 2020; Versace et al., 2015). Amygdala is a crucial region in the neural circuit involved in emotional competencies, and it is particularly vulnerable in the case of VPT birth (Johns et al., 2019). Additionally, altered amygdala connectivity in preterm children has been associated with social functioning deficits and emotional difficulties (Johns et al., 2019). The CB is another brain region that is particularly vulnerable in preterm children (Gano & Ferriero, 2017; Limperopoulos et al., 2014), and malformations have been linked to affective problems such as socialization deficits and mood abnormalities (Tavano et al., 2007). Diffusion MRI techniques have also demonstrated alterations in brain networks in preterm infants, particularly in the orbitofrontal cortex (Fischi‐Gómez et al., 2015; Rogers et al., 2012), which have been shown to correlate with specific emotional deficits at school age.

In contrast to the study by Nosarti et al. (2005), who reported reduced left caudate volume in association with hyperactivity behavior in ex‐preterm adolescent, we have not found an association between hyperactivity/inattention scores and brain volumes. These differences could be explained by the different ages at which brain volumes were measured and by the use of diverse methodologies for the delineation of the volumes.

Using the clinical dataset, we were able to predict the hyperactivity/inattention score with an AUC = 0.75. In that case, permutation importance analysis revealed that the most important variables in terms of classification accuracy of the model were sex, the presence of sepsis, and BPD.

Concerning the link between sex‐related differences and hyperactivity/inattention problems, post hoc analysis showed that females tend to have slightly higher scores, although the difference is not statistically significant. This is in‐line with several studies indicating that the male predominance of hyperactivity symptoms in the general population is missing in preterm children (Johnson & Marlow, 2011). Nevertheless, as Johnson and Marlow noted in their review (2011), most studies regarding gender‐related differences in mental outcomes among preterm children are mixed. Some research has shown that preterm boys are at higher risk for poorer social–emotional outcomes at school age (Samara et al., 2008), whereas other research has found a higher risk for withdrawal behavior in girls born VPT (Reijneveld et al., 2006).

Risk factors such as sepsis have been associated with adverse outcomes in preterm children (Lee et al., 2014), as well as a higher incidence of hyperactivity symptoms at preschool age (Kavas et al., 2017). In a study investigating behavioral abnormalities (measured by the Child Behavior Checklist) at 5 years of age in children born VPT in relation to neonatal morbidities, neonatal sepsis appeared to be a relevant risk factor for both internalizing and externalizing behavioral problems (Giordano et al., 2022). This is in‐line with our results, as the post hoc analysis showed a tendency for more hyperactivity at age 5 years in subjects who experienced neonatal sepsis. The impact of BPD on preterm infants’ long‐term outcomes covers a broad spectrum of consequences, including behavioral problems (Bora et al., 2011; Short et al., 2003). Our results suggest a slight association between BPD and hyperactivity/inattention symptoms. This association corroborates previous studies that reported a twofold of attention deficit/hyperactivity disorder in preterm children who suffered from BPD compared to controls at 8 years (Short et al., 2003). Consistent with our results, several studies did not find any significant relationship between behavioral problems at 5 years and the degree of prematurity (Yang et al., 2011).

When combining parental social economic risk with the volumetric and perinatal data, the prediction of the emotional symptoms score was achieved (AUC = 0.76). Permutation importance analysis unveiled that the volumetric and perinatal datasets were the most important in terms of the model's accuracy improvement. This is in‐line with the analysis’ results where only the volumetric data were used, which showed a good prediction for the emotional symptoms score. However, it is worth pointing out that in this analysis, where all three datasets were combined, the model's predictive ability was slightly higher (AUC = 0.76 for all three datasets, compared to AUC = 0.74 for the volumetric dataset alone). These findings are expected as the addition of more variables provides more information in terms of classification power. Somewhat surprising, in this model, the SES was the least important factor predicting the presence of emotional symptoms at 5 years. Family SES has been shown to be strongly related to cognitive outcome in preterm children (Bartal et al., 2020). Furthermore, the impact of social economic risk on the prevalence of behavioral, and more specifically, emotional problems, is well documented in typical children (Hosokawa & Katsura, 2018) and preterm populations (de Laat et al., 2016). In contrast to recent findings (Bartal et al., 2020), behavioral problems in this cohort are more linked to aberrant brain development and perinatal factors than environmental factors.

Finally, to better understand the influence of parental social economic risk on the behavioral outcome of the children born VPT, an LM on outcomes, including SES, volumetric data, and the perinatal risk factors, was fitted. Results indicate a significant positive correlation between the SES and the conduct problems score, and between SES and the peer problems score but not with any other behavioral score.

### 4.1 Limitations and future directions

Some limitations characterize the present study. First, the sample size was relatively modest. Because the SDQ is a screening test, and the small number of participants did not allow for separate consideration of the “borderline” and “abnormal” groups, we combined children from both groups into an “at risk” group, as we intended to include all children requiring further behavioral assessment. To screen for behavior problems more accurately in our cohort, we examined each subscale of the SDQ, although the number of children was very small, limiting the power available for statistical testing. Second, we were unable to compare our behavioral data with a group of 5‐year old children born at term, because these data were not available. Third, the classification performance might be influenced by the unbalanced nature of the two groups. However, to deal with the high specificity versus low sensitivity problem, stratified *K*‐fold cross‐validation was employed, and we reported results in terms of cross‐validated outcomes. Another limitation resides on the fact that the relations between volumetric brain data at TEA and behavioral outcome were investigated using global tissue volumes. Nevertheless, other studies explored these associations using more accurate brain segmentations providing more fine‐grained information on this relation (Anbeek et al., 2013).

Concerning the future directions, independent validation of our findings is needed to establish the robustness and reliability of the predictive biomarkers. In addition, recent studies highlighted the importance of other factors, such as exposure to painful experiences (Schneider, Duerden, et al., 2018) and nutritional deficit (Schneider, Fischer Fumeaux, et al., 2018), that appear to affect early brain maturation and, potentially, neurodevelopmental outcomes in VPT children. Future research should take these aspects into account, in order to have a more precise characterization of factors that can impact the long‐term development of children born too soon. Future use of predictive modeling could aim to identify novel potential predictive biomarkers of long‐term outcome. An accurate and reliable identification of brain and perinatal biomarkers can be extremely valuable as it would enable targeted early interventions aiming to address or even prevent the adverse outcomes observed in preterm‐born populations (e.g., by means of early life interventions).

## 5 CONCLUSIONS

This study is one of the few studies of VPT children looking at brain structure measured at TEA, and their relation to behavioral outcomes at 5 years of age. Brain volumes measured at TEA can contribute to the prediction of outcomes, but their relevance is limited to emotional symptoms. Perinatal clinical data represent good predictors of hyperactivity/inattention symptoms. Finally, higher parental social economic risk was related to more conduct and peer‐relations problems at 5 years of age. Based on these results, we can argue that predicting long‐term behavioral outcomes in VPT children is a complex task and various factors must be taken into account. The ability to better predict children's outcomes is crucial to set up early interventions aimed at improving prognosis and limit the detrimental impact of prematurity on children and families.

## AUTHOR CONTRIBUTIONS

Maria Chiara Liverani: data acquisition, conceptualization, writing‐original draft, writing‐review and editing. Serafeim Loukas: formal analysis, methodology, visualization, writing‐original draft, writing‐review and editing. Marie‐Pascale Pittet: conceptualization, writing‐review and editing. Laura Gui: methodology, writing‐review. Maricé Pereira: methodology, writing‐review. Anita C. Truttmann: data acquisition, writing‐review. Pauline Brunner: data acquisition. Myriam Bickle‐Graz: data acquisition, writing‐review. Petra S. Hüppi: writing‐review and editing, project administration, resources, funding acquisition. Djalel‐Eddine Meskaldji: data analysis, methodology, writing‐review, supervision. Cristina Borradori‐Tolsa: conceptualization, writing‐review, project administration, resources.

## CONFLICT OF INTEREST

The authors declare that they have no known competing financial interests or personal relationships that could have appeared to influence the work reported in this paper.

## FUNDING INFORMATION

Leenards Foundation under Grant Agreement Number: 2667 to P.S.H. The Swiss National Science Foundation under Grant Agreements Numbers: 33CM30_140334 and 32473B_135817.

### PEER REVIEW

The peer review history for this article is available at: https://publons.com/publon/10.1002/brb3.2818

## Data Availability

The data that support the findings of this study are available from the corresponding author upon reasonable request.
